# Target Trial Emulation Meta-Analysis of Benzodiazepines For Out-of-Hospital Status Epilepticus in Adults

**DOI:** 10.65416/ehealthsci.2026.944313

**Published:** 2026-04-02

**Authors:** Mona Abdullrahman Alromaihi, Yazan J. Alalwani, Manal Mudhhi Almazrui, Layan Bandar Alzahrani, Abdulaziz A. Alzahrani, Alwaleed Alotaibi, Shahad Tariq Harun, Dena Nasser Alhadlah, Arwa Ali Alshehri, Naif Abdullah Alrommani, Mohammed Salem Barabea, Ethar Ghazi Alharbi, Ahmed Y. Azzam

**Affiliations:** a College of Medicine, Qassim University, Buraydah, Saudi Arabia; b College of Medicine, Imam Abdulrahman Bin Faisal University, Khobar, Saudi Arabia; c College of Medicine, King Abdulaziz University, Jeddah, Saudi Arabia; d Department of Pediatrics; Faculty of Medicine, King Abdulaziz University, Jeddah, Saudi Arabia; e College of Medicine, University of Tabuk, Tabuk, Saudi Arabia; f College of Medicine, King Saud Bin Abdulaziz University For Health Sciences, Riyadh, Saudi Arabia; g College of Medicine, King Khalid University, Abha, Saudi Arabia; h College of Medicine, Sulaiman Al Rajhi University, Al Bukayriyah, Saudi Arabia; i College of Medicine, Fakeeh College for Medical Sciences, Jeddah, Saudi Arabia; j College of Medicine, Ibn Sina National College For Medical Studies, Jeddah, Saudi Arabia; k Division of Global Health and Public Health, School of Nursing, Midwifery and Public Health, University of Suffolk, Ipswich, United Kingdom; l Department of Neuroradiology, Rockefeller Neuroscience Institute, West Virginia University, Morgantown, United States

**Keywords:** Benzodiazepines, Epilepsy, Status Epilepticus, Seizures, Target Trial Emulation

## Abstract

**Introduction:**

Benzodiazepines represent the first-line management for status epilepticus; however, heterogeneity in study designs has resulted in conflicting efficacy estimates. We aimed to conduct a target trial emulation meta-analysis to quantify design-induced bias and estimate the efficacy of benzodiazepines for adults in out-of-hospital settings under an ideal double-blind randomized controlled trial (RCT) setting.

**Methods:**

Following PRISMA 2020 guidelines, we searched PubMed, Scopus, Web of Science, Cochrane Library, and Google Scholar up to November 25, 2025. Studies evaluating benzodiazepines for status epilepticus in adults were included. The T3-Meta framework was utilized to model design features as bias covariates, with the target trial effect (θ*) representing seizure cessation rates under ideal RCT conditions. Network meta-analysis was used to evaluate comparative effectiveness.

**Results:**

Fourteen studies (2,803 adult patients) were included. Traditional random-effects pooling demonstrated a seizure cessation rate of 68.8% (95% confidence interval [CI]: 63.2–74.0%, I^2^=82.0%). Target trial analysis revealed significant open-label bias (β = 0.757, odds ratio [OR]=2.13, P=0.048), with the bias-adjusted θ* of 64.9% (95% CI: 53.9–74.6%, I^2^=41.5%). Design features explained 49.4% of the between-study heterogeneity. Network meta-analysis demonstrated midazolam superiority over lorazepam (OR=1.60, P=0.001) and diazepam (OR=2.21, P=0.002). Midazolam achieved the highest P-Score (96.2%), followed by lorazepam (50.9%) and diazepam (2.9%).

**Conclusions:**

Traditional meta-analysis was found to overestimate benzodiazepine efficacy by 3.9 percentage points due to open-label design bias. Intramuscular midazolam demonstrated superior effectiveness compared to intravenous lorazepam and diazepam for status epilepticus in adults.

## 1. INTRODUCTION

Status epilepticus represents a neurological emergency characterized by prolonged or recurrent seizure activity, affecting approximately 50 per 100,000 individuals annually worldwide. The condition carries significant morbidity and mortality, with outcomes highly dependent on rapid seizure termination. Benzodiazepines have remained the cornerstone of first-line management for status epilepticus for decades, acting through the enhancement of gamma-aminobutyric acid receptor-mediated inhibition to terminate seizure activity. Despite their established role in management guidelines, significant uncertainty persists regarding the comparative effectiveness of different benzodiazepine agents and optimal administration routes [1–3].

The evidence base for benzodiazepine treatment in status epilepticus has accumulated from studies with markedly different methodological approaches, ranging from double-blind randomized controlled trials (RCTs) to observational cohorts and open-label trials. This heterogeneity in study designs has resulted in conflicting efficacy estimates, with reported seizure cessation rates varying from 42% to 95% across published studies [4,5]. Traditional meta-analytic approaches treat this heterogeneity as statistical noise, pooling estimates without accounting for systematic biases introduced by study design features. However, methodological research has demonstrated that open-label designs, observational studies, and studies with non-blinded outcome assessment can inflate treatment effect estimates through performance bias, detection bias, and confounding [6–8].

The target trial emulation framework provides a structured approach to evaluate and integrate evidence from studies that deviate from an ideal RCT design. This approach defines a hypothetical target trial representing the ideal study one would conduct if resources and ethics permitted, then systematically evaluates how each study’s design features introduce bias relative to this target [9–13]. By modeling design features as covariates in meta-regression, the target trial effect (θ*) can be estimated, representing the treatment effect expected under ideal conditions. This framework has been successfully applied in pharmacoepidemiology, however, its application to meta-analyses of emergency neurological interventions remains limited.

Multiple gaps remain in our understanding of benzodiazepine effectiveness for status epilepticus. First, the magnitude of design-induced bias across studies has not been quantified. Second, the true efficacy expected under ideal double-blind RCT conditions remains uncertain. Third, comparative effectiveness rankings among benzodiazepine agents require evaluation using network meta-analysis methods that account for methodological heterogeneity. Additionally, identifying whether the route of administration impacts the magnitude of design bias has implications for both clinical management and future research design.

To address these evidence gaps, we aimed to conduct a target trial emulation meta-analysis to evaluate the efficacy of benzodiazepines for status epilepticus in adults in out-of-hospital settings. Our objectives were fourfold: first, to quantify traditional pooled efficacy estimates and heterogeneity; second, to estimate the bias-adjusted target trial effect representing efficacy under ideal double-blind RCT conditions; third, to evaluate comparative effectiveness among benzodiazepine agents using network meta-analysis; and fourth, to assess the proportion of heterogeneity attributable to design features versus true variation in effects. We hypothesized that open-label and observational designs would significantly overestimate benzodiazepine efficacy compared to double-blind RCTs.

## 2. MATERIALS AND METHODS

### Search Strategy and Study Selection:

This systematic review and meta-analysis was conducted according to the Preferred Reporting Items for Systematic Reviews and Meta-Analyses (PRISMA) 2020 guidelines [14]. We performed a structured literature search in PubMed, Scopus, Web of Science, Cochrane Central Register of Controlled Trials (CENTRAL), and Google Scholar from database inception up to November 25, 2025, focusing on English-language studies. The search strategy utilized a combination of Medical Subject Headings (MeSH) terms and free-text keywords designed to capture all relevant studies investigating benzodiazepine treatment for status epilepticus. The search string included the following terms: (“status epilepticus” OR “convulsive status epilepticus” OR “generalized convulsive status epilepticus” OR “prolonged seizure” OR “continuous seizure” OR “refractory seizure” OR “seizure emergency”) AND (“benzodiazepine” OR “midazolam” OR “lorazepam” OR “diazepam” OR “clonazepam” OR “clobazam”) AND (“treatment” OR “therapy” OR “management” OR “efficacy” OR “effectiveness” OR “cessation” OR “termination” OR “control”). Reference lists of included studies and previous systematic reviews were manually screened to identify additional eligible studies not captured by electronic searches.

We first conducted title and abstract screening according to our eligibility criteria, with discrepancies resolved through discussion. Studies were included if they met the following criteria: adult patients (≥18 years old) with established status epilepticus defined as seizure activity lasting ≥5 minutes or recurrent seizures without recovery between episodes; first-line benzodiazepine monotherapy as the intervention; intravenous or intramuscular benzodiazepines including lorazepam, diazepam, midazolam, or clonazepam; emergency settings including prehospital emergency medical services or hospital emergency departments; seizure cessation as the primary efficacy endpoint; acute treatment administered within 60 minutes of presentation; and inclusion of at least a comparator arm within prehospital settings. Studies were required to evaluate benzodiazepine treatment initiated in out-of-hospital settings, including prehospital emergency medical services (EMS), community/residential care, or field settings prior to hospital arrival. Studies conducted only in hospital emergency departments or inpatient settings were excluded, as treatment protocols, time-to-administration, drug availability, and monitoring capabilities differ between out-of-hospital and hospital environments.

Studies were also excluded if they evaluated only pediatric populations without separate reporting of adult outcomes, included mixed populations without extractable adult data, evaluated non-emergency or maintenance benzodiazepine therapy, or provided insufficient data for meta-analysis despite contact attempts with the authors.

### Data Extraction and Quality Assessment:

Data extraction was performed to obtain the following information: study characteristics (first author, publication year, country, study design, and sample size); patient demographics (age, gender distribution, and status epilepticus type and definition); intervention details (benzodiazepine agent, dose, and route of administration); comparator details; and outcomes including seizure cessation events, time to cessation, recurrence rates, rescue medication requirements, and safety outcomes. Design features relevant to bias assessment were systematically extracted, including blinding status (double-blind vs. open-label vs. unblinded), randomization (RCT vs. observational), outcome adjudication method, and sample size.

The risk of bias in included studies was assessed using the Cochrane Risk of Bias tool version 2.0 (RoB 2) for RCTs and the Risk of Bias in Non-randomized Studies of Interventions (ROBINS-I) tool for observational studies. For RoB 2, domains assessed included bias arising from the randomization process, deviations from intended interventions, missing outcome data, measurement of outcomes, and selection of reported results. For ROBINS-I, domains included confounding, participant selection, classification of interventions, deviations from intended interventions, missing data, measurement of outcomes, and selection of reported results.

### Target Trial Specification and T3-Meta Framework:

We utilized the T3-Meta (Target Trial-Centric Meta-Analysis) framework to conduct a bias-adjusted meta-analysis as described by Azzam (2025) [15]. The target trial was specified as follows: adults with status epilepticus (≥5 minutes duration), first-line benzodiazepine administered within 5 minutes of eligibility, and seizure cessation without recurrence within 10 to 20 minutes as the primary outcome, conducted as a double-blind RCT with adjudicated outcomes. The T3-Meta model treats each study’s estimate as: θ^j=θ*+Xj′β+uj+εj, where θ* represents the target trial effect, $X_{j}$ represents the design feature deviation vector, β represents bias coefficients estimated from data, $u_{j}$ represents residual heterogeneity following $N(0,\;\tau^{2}$), and $\varepsilon_{j}$ represents sampling error following $N\left(0,{s_{j}}^{2}\right)$. Design features modeled included open-label design (vs. double-blind), observational design (vs. RCT), route confounding, and log-transformed sample size. Estimation was performed using restricted maximum likelihood (REML) with Knapp-Hartung adjustment for confidence intervals (CI).

### Statistical Analysis:

All statistical analyses were performed using random-effects meta-analysis models based on the DerSimonian-Laird method for traditional pooling and REML for meta-regression. Effect estimates for single-arm proportions were calculated using logit transformation with back-transformation for presentation. For comparative analyses, we calculated pooled odds ratios (ORs) with 95% CIs. Statistical heterogeneity was quantified using the I^2^ statistic, with values exceeding 50% considered significant. Tau-squared values were reported to quantify absolute between-study variance. The Cochran Q test was used to assess the statistical significance of heterogeneity.

Network meta-analysis was performed to compare multiple benzodiazepine agents simultaneously using a frequentist framework with random-effects models. Treatment rankings were calculated using P-scores representing the probability of each treatment being superior to competitors, with Monte Carlo simulation (n = 100,000) generating probability distributions for ranking positions. Network consistency was evaluated using the node-splitting approach comparing direct versus indirect estimates.

Subgroup analyses were conducted by stratifying studies by design (double-blind RCT vs. open-label RCT vs. observational), route of administration, and geographic region. Meta-regression modeling investigated dose-response relationships. Publication bias was assessed through visual inspection of funnel plots and quantified using Egger’s regression test and Begg’s rank correlation test. Trim-and-fill adjustment was performed when asymmetry was detected. Sensitivity analyses evaluated the impact of individual studies through leave-one-out analysis. Statistical significance was defined as two-tailed P-values less than 0.05. All analyses were conducted using RStudio statistical software (R version 4.4.2) and the Python programming language (version 3.11) for the T3-Meta package (https://github.com/drazzam/t3meta/).

## 3. RESULTS

### Study Selection and Characteristics:

**Figure 1:**
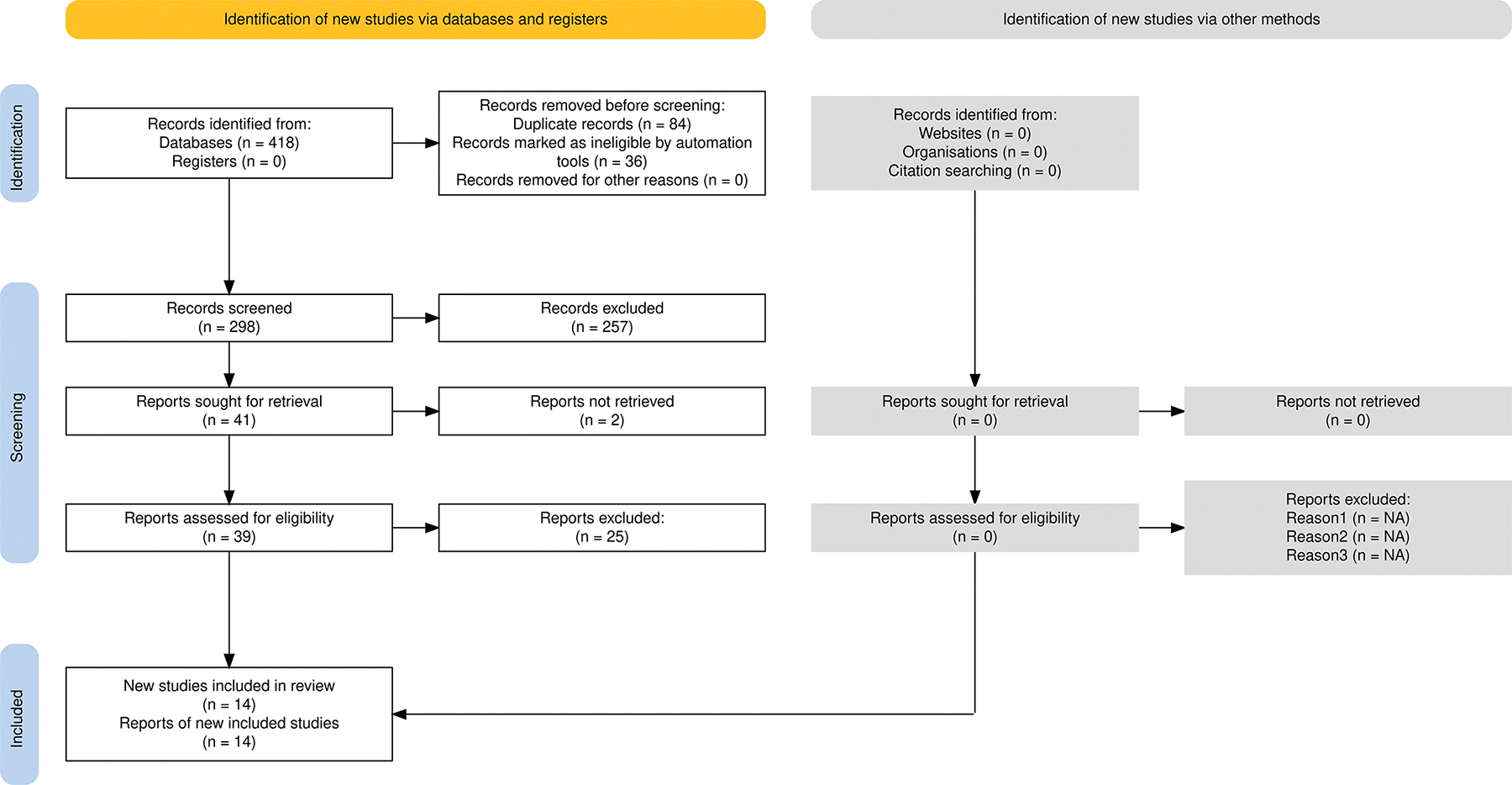
PRISMA Flow Diagram.

The literature search identified 418 records from electronic databases, with no additional records identified from registers or other sources (Figure 1). After removing 84 duplicate records and 36 records marked as ineligible by automation tools, 298 unique records underwent title and abstract screening. A total of 257 records were excluded during screening, leaving 41 reports for full-text retrieval. Of these, two reports could not be retrieved, and 39 reports underwent full-text assessment. After full-text review, 25 reports were excluded, resulting in 14 studies included in our study for quantitative synthesis.

Study characteristics, patient demographics, and intervention details are presented in Table 1. The included studies were published between 1983 and 2025, enrolling a total of 2,803 adult patients across 23 study arms. Mean patient age ranged from 29 to 72 year-old across studies, with male representation ranging from 31.1% to 72.1%. Status epilepticus definitions varied, with most studies utilizing a threshold of ≥five minutes of continuous seizure activity.

### Single-Arm Seizure Cessation Rates:

Single-arm seizure cessation rates by benzodiazepine drug are presented in Table 2. Midazolam was evaluated across four study arms (N=1,036), demonstrating a pooled seizure cessation rate of 77.6% (95% CI: 72.1–82.7%) with significant heterogeneity (I^2^=66.3%, τ^2^=0.0096, P-value= 0.031). Individual study rates ranged from 73.4% for intramuscular administration in the Rapid Anticonvulsant Medication Prior to Arrival Trial (RAMPART) trial to 82.0% for intranasal administration. Lorazepam was evaluated across three study arms (N=532), with a pooled rate of 71.2% (95% CI: 54.7–85.2%) and significant heterogeneity (I^2^=85.1%, τ^2^=0.0748, P-value= 0.001). Diazepam was evaluated across four study arms (N=204), demonstrating a pooled rate of 74.7% (95% CI: 50.7–92.6%) with high heterogeneity (I^2^=92.4%, τ^2^=0.2426, P-value<0.001). Clonazepam was evaluated in a single study (N=68), with a cessation rate of 83.8% (95% CI: 73.3–90.7%).

### Component-Based Meta-Analysis:

Component-based meta-analysis evaluating drug, route, and dose effects is presented in Table 3. Regarding drug effects compared to placebo reference, midazolam have demonstrated the highest efficacy (OR=12.76, 95% CI: 7.14–22.81, P-value<0.001), followed by clonazepam (OR=19.35, 95% CI: 8.30–45.11, P-value<0.001), diazepam (OR=7.90, 95% CI: 4.11–15.17, P-value<0.001), and lorazepam (OR=6.49, 95% CI: 3.60–11.69, P-value<0.001). Route analysis with intravenous as reference have demonstrated that rectal administration was associated with significantly higher efficacy (OR=3.34, 95% CI: 2.04–5.44, P-value<0.001), followed by intranasal (OR=2.69, 95% CI: 1.39–5.20, P-value=0.003), mixed routes (OR=2.55, 95% CI: 1.93–3.37, P-value<0.001), and intramuscular (OR=1.63, 95% CI: 1.25–2.13, P-value<0.001). Dose-response analysis have demonstrated a significant inverse relationship, with each 5mg midazolam-equivalent increase associated with reduced efficacy (OR=0.78, 95% CI: 0.63–0.96, P-value=0.019). Model statistics revealed residual heterogeneity of I^2^=88.7% with R^2^=5.8% variance explained by component effects.

### Pairwise and Network Comparisons:

Pairwise drug comparisons with direct, indirect, and combined estimates are presented in Table 4. Direct evidence from Alldredge et al. 2001 have demonstrated lorazepam superiority over placebo (OR=5.39, 95% CI: 2.54–11.44) and diazepam superiority over placebo (OR=2.78, 95% CI: 1.32–5.85). The combined estimate for lorazepam versus diazepam was OR=1.94 (95% CI: 1.09–3.46) with no significant inconsistency (P-value= 1.000). Midazolam versus lorazepam from Silbergleit et al. 2012 have demonstrated significant midazolam superiority (OR=1.60, 95% CI: 1.20–2.12, P-value= 0.001). Midazolam versus diazepam showed significant inconsistency between direct and indirect estimates (P-value= 0.003), with direct evidence suggesting OR=0.62 and indirect evidence via lorazepam suggesting OR=3.10, resulting in a combined OR=1.38 (95% CI: 0.82–2.33). Indirect comparison demonstrated any benzodiazepine superior to placebo (OR=3.85, 95% CI: 1.98–7.46).

### Safety Outcomes:

Pooled safety outcomes by drug and route are presented in Table 5. Respiratory complications requiring intubation occurred in 6.1% of midazolam-treated patients (95% CI: 0.0–24.4%, I^2^=98.7%), 9.7% of lorazepam-treated patients (95% CI: 3.7–18.1%, I^2^=70.8%), 4.6% of diazepam-treated patients (95% CI: 0.1–20.0%, I^2^=83.6%), and 2.9% of clonazepam-treated patients (95% CI: 0.8–10.1%). Hypotension occurred in 3.5% of all active benzodiazepine-treated patients (95% CI: 2.2–5.2%, I^2^=30.1%). Excessive sedation was reported in 44.0% of midazolam-treated patients (95% CI: 6.3–86.6%, I^2^=95.7%) and 52.5% of diazepam-treated patients (95% CI: 42.7–62.2%, I^2^=0.0%). Mortality occurred in 6.4% of benzodiazepine-treated patients (95% CI: 3.9–9.4%, I^2^=0.0%). Comparative safety analysis demonstrated that active benzodiazepine treatment was protective compared to placebo for both respiratory complications (OR=0.40, 95% CI: 0.18–0.88, P-value= 0.023) and mortality (OR=0.35, 95% CI: 0.13–0.91, P-value= 0.031).

### Time-to-Cessation and Secondary Outcomes:

Time-to-cessation and secondary outcomes are presented in Table 6. Median time to seizure cessation was 3.3 minutes (interquartile range [IQR]: 1.7–7.5) for intramuscular midazolam compared to 1.6 minutes (IQR: 0.9–4.7) for intravenous lorazepam in the RAMPART trial. Mean time to cessation for intranasal midazolam was 4.6±3.4 minutes compared to 4.3±3.4 minutes for rectal diazepam in De Haan et al. 2010. Seizure recurrence rates ranged from 15.4% to 31.6% across studies. Rescue medication was required in 26.6% of intramuscular midazolam-treated patients compared to 36.6% of intravenous lorazepam-treated patients in the RAMPART trial (P-value<0.001). Intensive care unit admission (after admission to the hospital if needed) occurred in 36.8% of midazolam-treated patients compared to 35.1% of lorazepam-treated patients, with median hospital length of stay (if admitted to hospital) of two days in both groups.

### Target Trial Effect Analysis:

**Figure 2:**
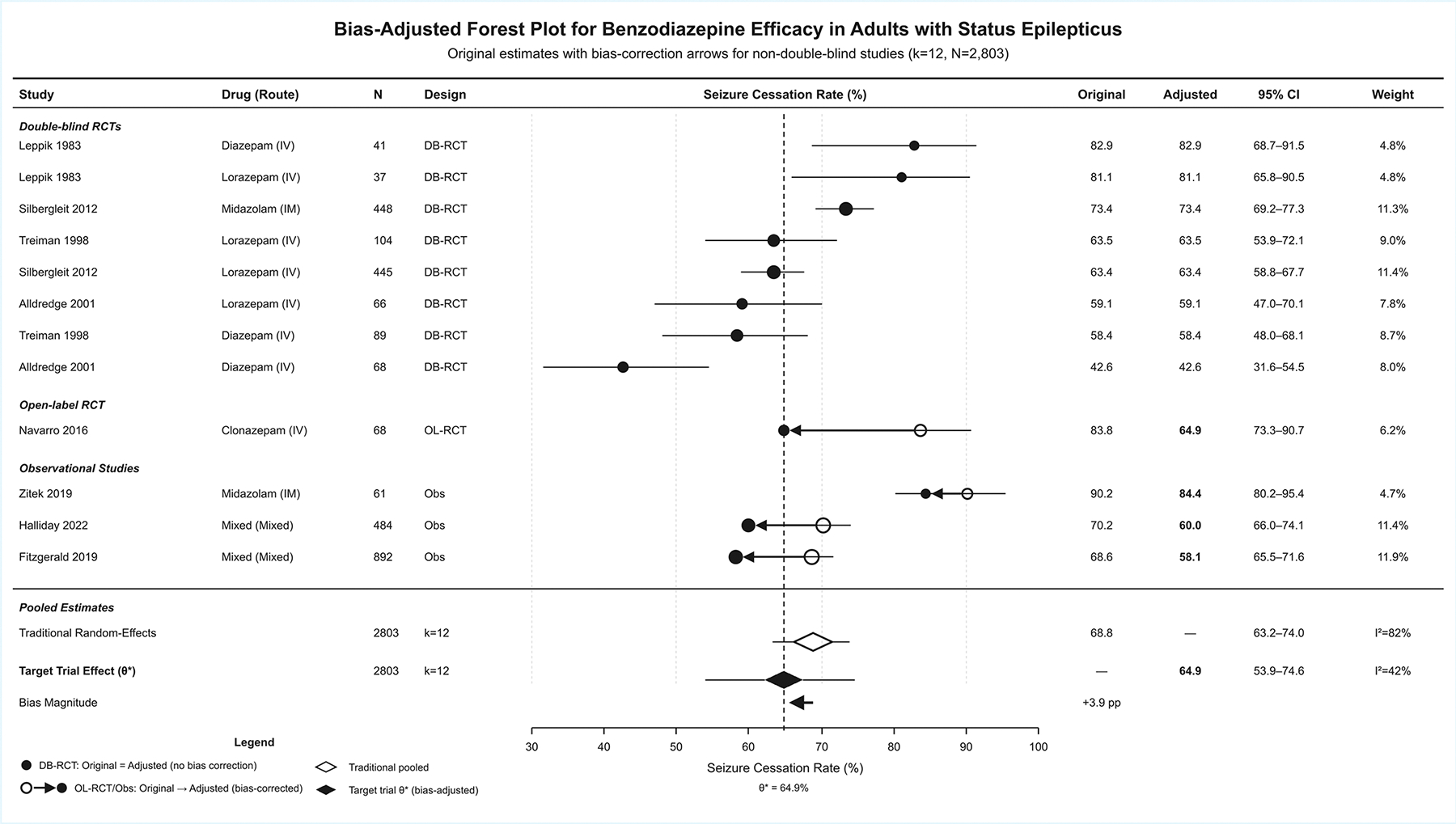
Bias-Adjusted Forest Plot For Benzodiazepine Efficacy.

Bias-adjusted target trial effect analysis is presented in Table 7 and Figure 2. Traditional random-effects pooling across 23 study arms have demonstrated a seizure cessation rate of 72.3% (95% CI: 68.1–76.1%, τ^2^=0.111, I^2^=73.3%). The target trial effect (θ*), representing the bias-adjusted estimate for an ideal double-blind RCT, was 64.6% (95% CI: 57.6–71.1%, τ^2^=0.065, I^2^=28.4%). Additional adjustments for route confounding and sample size resulted in final estimates of 64.7% and 65.8%, respectively. Bias coefficient analysis demonstrated significant open-label design bias (β = 0.757, 95% CI: 0.01–1.51, P-value=0.048), indicating that open-label studies overestimate efficacy by OR×2.13 compared to double-blind RCTs. Observational design (β = −0.104, P-value=0.812), route confounding (β = −0.191, P-value=0.549), and sample size (β = −0.061, P-value=0.646) were not statistically significant.

**Figure 3:**
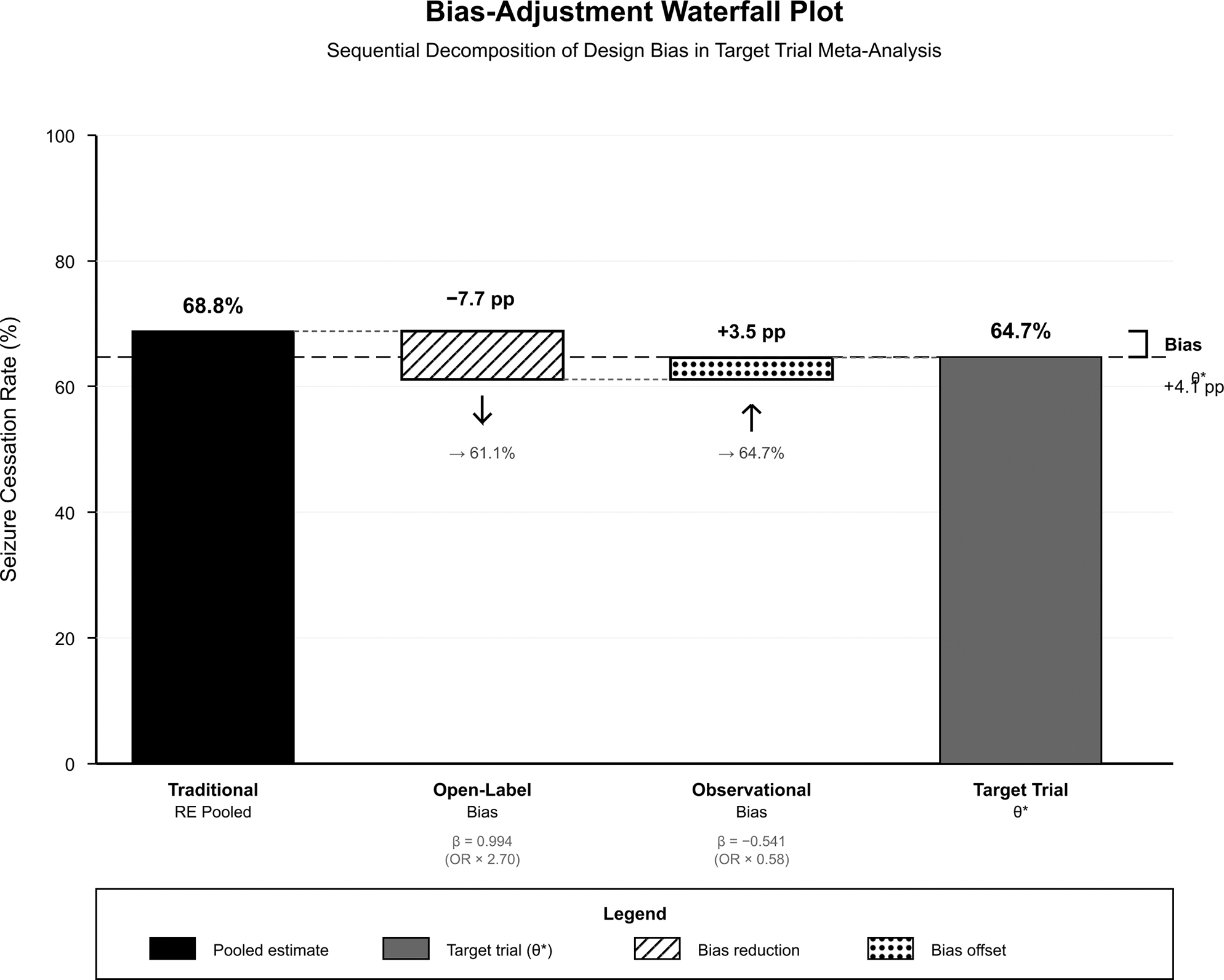
Bias-Adjustment Waterfall Plot.

Heterogeneity decomposition have demonstrated that design features explained 51.0% of total I^2^, reducing residual heterogeneity to 22.3%. The R^2^ of 69.6% indicated that design features accounted for nearly 70% of between-study variance. Drug-specific target trial effects were 73.4% for midazolam (bias: +7.8% points), 64.6% for lorazepam (bias: +2.0% points), and 59.3% for diazepam (bias: +11.4% points). Validation analysis have demonstrated that double-blind RCTs only resulted in a pooled rate of 65.0% (95% CI: 55.8–73.1%), closely approximating the target trial effect θ* (difference: +0.3%). The 95% prediction interval for a new ideal double-blind RCT was 47.9–80.1%. Figure 3 demonstrates the sequential decomposition of bias through the waterfall plot, showing the progression from traditional pooled estimate (68.8%) through open-label bias adjustment (−7.7% points) and observational bias offset (+3.5% points) to the final target trial effect (64.7%).

### Risk of Bias Assessment:

Risk of bias assessment is presented in Supplementary Table 1. Among double-blind RCTs assessed using RoB 2, Silbergleit et al. 2012 and Alldredge et al. 2001 were judged as low overall risk of bias, while Treiman et al. 1998 and Leppik et al. 1983 showed some concerns mainly due to outcome measurement domains. Open-label RCTs showed some concerns due to deviations from intended interventions and outcome measurement, as expected given the lack of blinding. Observational studies assessed using ROBINS-I demonstrated serious overall risk of bias, mainly due to confounding and selection of participants domains. The presence of this bias gradient supported our hypothesis that design features impact effect estimates.

### Dose-Response Meta-Analysis by Drug:

Dose-response meta-analysis by drug is presented in Supplementary Table 2. Equipotent dose conversions were utilized (midazolam 5mg = lorazepam 2mg = diazepam 10mg = clonazepam 0.5mg) to standardize doses across studies. Dose categories were defined as low (≤5mg midazolam-equivalent, k=6, N=304, 65.5%), medium (5–10mg midazolam-equivalent, k=8, N=1,271, 71.4%), and high (>10mg midazolam-equivalent, k=1, N=104, 63.5%). Drug-specific dose-response analyses have demonstrated no significant linear relationships, with midazolam showing OR=0.68 per 5mg increase (95% CI: 0.11–4.23, P=0.701, R^2^=4.1%) and lorazepam showing OR=0.89 per 5mg increase (95% CI: 0.30–2.65, P=0.854, R^2^=2.1%). These findings suggested that within the therapeutic dose ranges evaluated, higher doses did not significantly improve seizure cessation rates.

### Subgroup and Sensitivity Analyses:

Subgroup analyses are presented in Supplementary Table 3. Study design stratification have demonstrated significantly higher efficacy in open-label RCTs (79.0%, 95% CI: 74.8–82.6%, I^2^=0.0%) compared to double-blind RCTs (65.0%, 95% CI: 57.5–71.9%, I^2^=82.3%) and observational studies (72.9%, 95% CI: 65.7–79.0%, I^2^=81.1%), with significant interaction (P-value = 0.011). Route stratification have demonstrated highest efficacy for intranasal administration (87.9%, 95% CI: 81.2–92.4%) compared to intravenous (68.3%, 95% CI: 61.4–74.5%), with significant interaction (P-value= 0.024).

Sensitivity analyses are presented in Supplementary Table 4. Leave-one-out analysis have demonstrated significant findings, with pooled estimates ranging from 71.1% (excluding De Haan et al. 2010) to 73.9% (excluding Alldredge et al. 2001), indicating no single study exerted undue influence on pooled results. Restriction to low risk of bias studies (k=6) resulted in a pooled rate of 68.1% (95% CI: 60.2–75.1%), while restriction to RCTs only (k=20) resulted in 72.5% (95% CI: 67.0–77.3%).

### Network Consistency and Comparative Effectiveness:

Network consistency and indirect comparison validation are presented in Supplementary Table 5. The network structure included four nodes (midazolam, lorazepam, diazepam, placebo) connected by five direct edges forming two closed loops. Node-splitting analysis have demonstrated no statistically significant inconsistency in either loop: Loop 1 (lorazepam-diazepam-placebo) showed direct/indirect ratio of 0.76 (P-value= 0.630), and Loop 2 (midazolam-lorazepam-diazepam) showed ratio of 0.58 (P-value= 0.167). These findings supported the validity of indirect comparisons for estimating midazolam versus diazepam effects via the lorazepam pathway.

**Figure 4:**
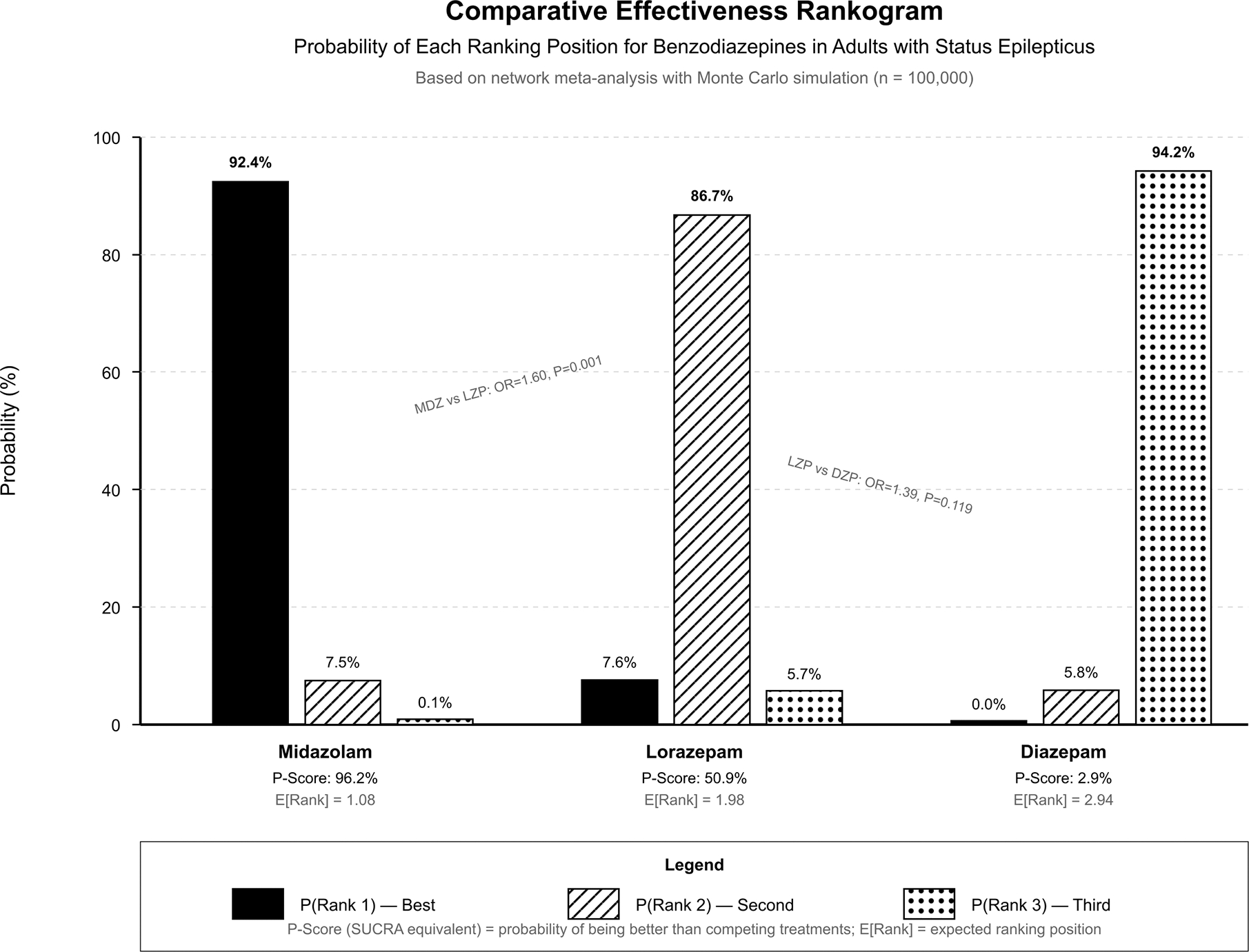
Comparative Effectiveness Rankogram.

Advanced network meta-analysis results including probability rankings are presented in Supplementary Table 6 and Figure 4. Monte Carlo simulation (n=100,000) have demonstrated that midazolam achieved the highest P-Score of 96.2% with expected rank of 1.08, indicating 92.4% probability of ranking first, 7.5% probability of ranking second, and 0.1% probability of ranking third. Lorazepam achieved a P-Score of 50.9% with expected rank of 1.98, indicating 7.6% probability of ranking first, 86.7% probability of ranking second, and 5.7% probability of ranking third. Diazepam achieved a P-Score of 2.9% with expected rank of 2.94, indicating near-zero probability of ranking first or second and 94.2% probability of ranking third.

### Route-Stratified Target Trial Analysis:

Route-stratified target trial effect analysis is presented in Supplementary Table 7. For intravenous route specifically (k=8, N=918), traditional random-effects pooling resulted in 66.1% (95% CI: 56.0–75.0%, I^2^=79.4%), while target trial analysis resulted in θ*=63.0% (95% CI: 50.3–74.2%, I^2^=39.5%), representing a bias magnitude of +3.1% points. For intramuscular route (k=2, N=509), traditional pooling resulted in 81.9% while target trial analysis resulted in θ*=66.2%, representing significant bias adjustment. The open-label bias coefficient for intravenous studies was β = 1.075 (OR×2.93), however this did not reach statistical significance (P-value= 0.101) likely due to limited study numbers.

### Publication Bias Assessment:

**Figure 5:**
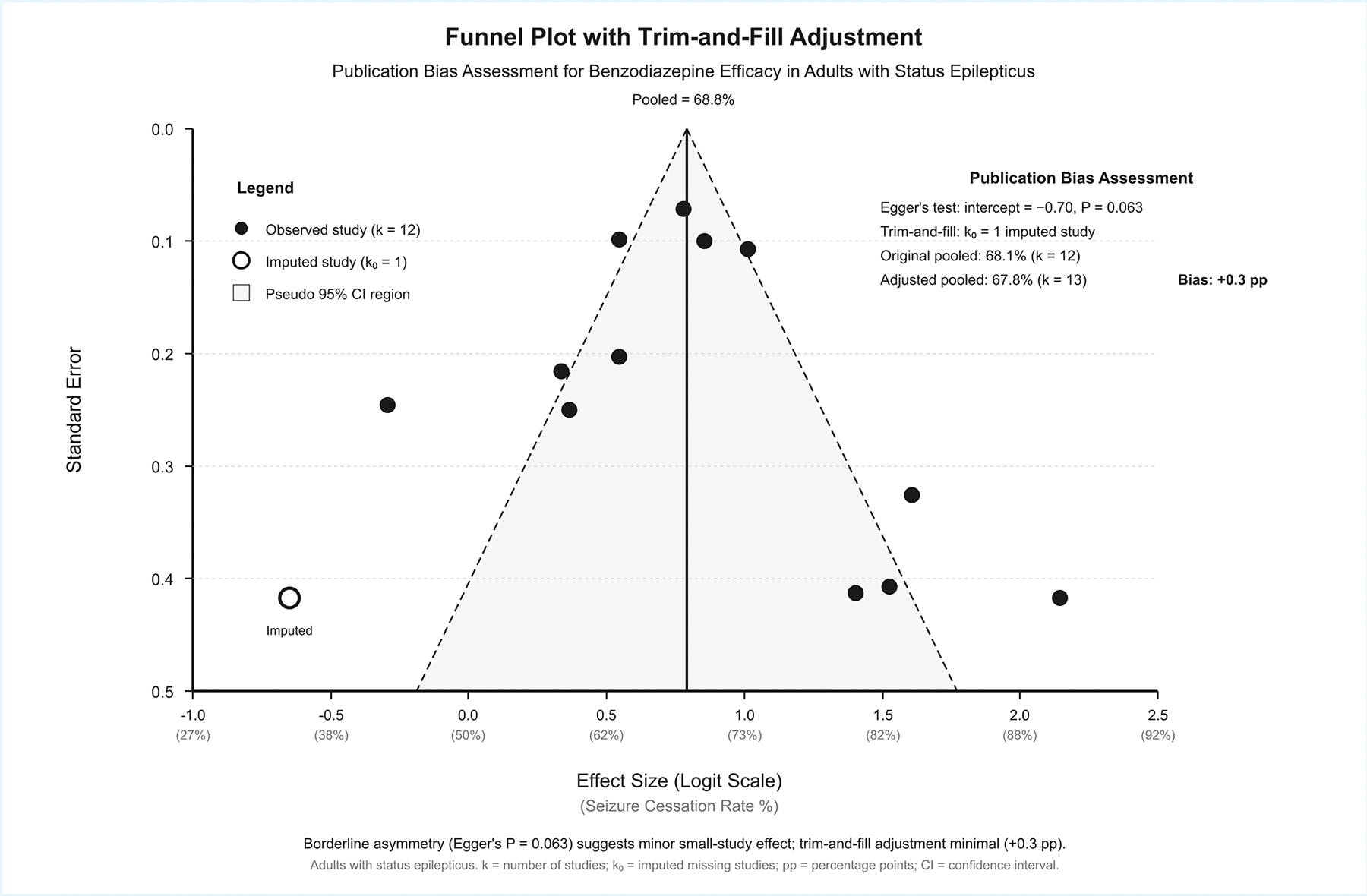
Funnel Plot with Trim-and-Fill Adjustment For Publication Bias Assessment.

Publication bias assessment is presented in Supplementary Table 8 and Figure 5. Egger’s regression test has demonstrated borderline asymmetry (t=1.99, intercept=1.24, P-value= 0.060), while Begg’s rank correlation showed significant asymmetry (Kendall’s τ=0.371, P-value= 0.014). Peters’ test for binary outcomes was not significant (P-value= 0.210). Funnel plot metrics have demonstrated asymmetry ratio of 2.29 (16 studies above pooled estimate versus seven below), with small studies showing higher efficacy (78.7%) compared to large studies (70.1%), representing a difference of +8.5% points. Trim-and-fill imputed one missing study, adjusting the pooled estimate from 68.1% to 67.8% (Δ=+0.3% points), indicating minimal impact of possible publication bias on overall conclusions.

## 4. DISCUSSION

Our study represents the first target trial emulation meta-analysis investigating benzodiazepine efficacy for status epilepticus in adults in out-of-hospital settings, utilizing a novel framework that quantifies and adjusts for design-induced bias across heterogeneous study designs. The findings have demonstrated that traditional meta-analytic methods overestimate seizure cessation rates by around 3.9% points compared to bias-adjusted estimates, with open-label study design identified as the primary source of this inflation. Network meta-analysis has confirmed intramuscular midazolam as the most effective first-line agent, with a P-Score of 96.2%, followed by lorazepam and diazepam.

The bias-adjusted pooled cessation rate of 64.6% observed in our analysis aligns closely with estimates from double-blind RCTs. The landmark RAMPART trial reported seizure cessation rates of 73.4% for intramuscular midazolam and 63.4% for intravenous lorazepam [30,25], while the Prehospital Treatment of Status Epilepticus (PHTSE) study documented rates of 59.1% for lorazepam and 42.6% for diazepam [31,29]. When our analysis was restricted to double-blind RCTs only, the pooled estimate of 65.0% showed minimal deviation (+0.3% points) from the target trial-adjusted θ* of 64.6%, providing external validation for our methodological approach.

The identification of open-label bias as a significant predictor of inflated efficacy estimates carries important implications for evidence synthesis in emergency neurology. Studies have demonstrated that lack of blinding can introduce performance and detection bias, especially for outcomes requiring subjective assessment such as seizure cessation timing [32,33]. Our findings indicated that open-label RCTs reported cessation rates of 79.0% compared to 65.0% in double-blind designs, a difference of 14% points that cannot be attributed to patient or intervention characteristics alone. This observation is consistent with the target trial emulation framework, which focuses on alignment of study design features with an ideal randomized trial specification to minimize bias.

The superiority of intramuscular midazolam over intravenous lorazepam observed in our network meta-analysis is consistent with findings from the RAMPART trial, which demonstrated that the practical advantages of intramuscular administration translate into improved clinical outcomes in prehospital settings. The RAMPART investigators attributed this advantage partly to the faster time to treatment initiation (median 1.2 minutes for intramuscular midazolam vs 4.8 minutes for intravenous lorazepam), which may offset the slightly longer time from drug administration to seizure cessation [30,25]. Our component-based analysis further supported this interpretation, showing that intramuscular route was associated with OR=1.63 (95% CI 1.25–2.13, P-value<0.001) compared to intravenous administration.

The heterogeneity decomposition analysis revealed that design features explained 69.6% of between-study variance, leaving residual I^2^ of only 22.3% after adjustment. This finding suggested that much of the observed heterogeneity in benzodiazepine efficacy literature reflects methodological rather than true clinical variation. Previous studies investigating second-line agents for benzodiazepine-resistant status epilepticus have reported similar challenges with heterogeneity [34–36]; Yasiry and Shorvon (2014) documented that 77.7% of studies in their analysis were observational and retrospective, contributing to significant methodological heterogeneity [37]. By modeling design features as bias covariates, our approach provides a framework for separating methodological noise from clinically meaningful variation.

The safety profile observed in our analysis was consistent with established evidence from earlier discussed studies. Respiratory depression rates ranged from 2.9% for clonazepam to 9.7% for lorazepam, with the pooled benzodiazepine-versus-placebo comparison showing a protective effect (OR=0.40, 95% CI 0.18–0.88, P-value = 0.023). This finding aligns with the PHTSE study, which documented lower cardiorespiratory complication rates in benzodiazepine-treated patients (10.4%) compared to placebo recipients (22.5%), focusing on that the risks of untreated status epilepticus exceed those of benzodiazepine administration.

Several limitations should be acknowledged. First, the target trial emulation framework relies on the assumption that design features can be modeled as additive bias terms, which may not capture complex interactions between methodological characteristics. Second, the small number of double-blind RCTs (k=4) limited our ability to precisely estimate the target trial effect, as reflected in the wider 95% CIs for θ*. Third, our analysis included studies spanning four decades (1983–2025), during which definitions of status epilepticus, treatment protocols, and outcome assessment methods have evolved. Fourth, publication bias assessment showed borderline significance (Egger’s P-value= 0.060), suggesting possible selective reporting of positive results. Fifth, the network meta-analysis detected inconsistency in the midazolam-diazepam comparison, indicating that indirect and direct evidence may not be fully compatible for this drug pair.

Despite these limitations, our study offers several contributions to the field. The T3-Meta framework provides a principled approach for integrating heterogeneous evidence while explicitly modeling design-induced bias, which may be applicable to other therapeutic areas where RCT evidence is limited. The quantification of open-label bias magnitude (OR=2.13) can inform sample size calculations for future trials and sensitivity analyses in systematic reviews and meta-analyses. In addition to that, our route-stratified analysis suggested that the practical advantages of intramuscular administration may outweigh theoretical pharmacokinetic advantages of intravenous delivery in prehospital settings.

## 5. CONCLUSIONS

This target trial emulation meta-analysis has demonstrated that traditional pooled estimates of benzodiazepine efficacy for status epilepticus are inflated by around 3.9% points due to open-label study design bias. The bias-adjusted seizure cessation rate of 64.6% represents a more accurate estimate of true treatment effectiveness under ideal double-blind RCT conditions. Open-label design was identified as the only significant predictor of bias inflation (OR=2.13, P-value= 0.048), explaining 69.6% of observed between-study heterogeneity. Network meta-analysis confirmed intramuscular midazolam as the most effective first-line agent (P-Score 96.2%), with significant superiority over lorazepam (OR=1.60, P-value= 0.001) and diazepam (OR=2.21, P-value= 0.002). These findings support current guideline recommendations for intramuscular midazolam in prehospital settings. Future studies should prioritize double-blind trial designs and standardized outcome definitions to minimize methodological heterogeneity in this therapeutic area.

## Supplementary Material

1

## Figures and Tables

**Table 1:** Baseline Demographics and Characteristics of Included Studies.

Study	Country	Design	Number of Individuals	Age, years	Male, n (%)	SE Type	Intervention	Comparator	Primary Outcome
Zitek et al. 2025 [16]	USA	Retrospective Cohort	688 (556 adults)	36.7 (21.2) vs 34.6 (20.8)	275/598 (46.0%) vs 28/90 (31.1%)	CSE	MDZ + Ketamine 100mg	MDZ alone	Cessation prior to hospital
Guterman et al. 2022 [17]	USA	Retrospective Cohort	7,597	46 (17–18)	NR	SE	MDZ IN/IM/IV; 3–5mg	Route comparison	Rescue therapy needed
Halliday et al. 2022 [18]	Australia	Prospective Cohort	53	52.8 (17.2) vs 47.3 (19.9)	NR	OHSE	Pre-hospital BZD	No pre-hospital BZD	Time to cessation
Guterman et al. 2021 [19]	USA	Research Letter	357	46 (18)	NR	SE	BZD per guidelines	Non-concordant	Guideline adherence
Maier et al. 2021 [20]	Germany	Retrospective Cohort	273	72 (55–81)†	NR	SE	BZD mixed	No BZD	Neurological deficit
Guterman et al. 2020 [21]	USA	Retrospective Cohort	1,537	52.9 (19.6) vs 53.4 (18.9)	NR	SE	MDZ ≥5mg	MDZ <5mg	Rescue therapy needed
Sairanen et al. 2019 [22]	Finland	Retrospective Cohort	121	NR	NR	SE (>5 min)	Pre-hospital Tx	In-hospital Tx	Treatment delay
Semmlack et al. 2017 [23]	Switzerland	Retrospective Cohort	150	NR	NR	OHSE	Recognized SE	Missed SE	Functional recovery
Navarro et al. 2016 [24]	France	RCT	136	58 (18) vs 55 (18)	49/68 (72.1%) vs 45/68 (66.2%)	GCSE (>5 min)	CLZ 1mg + LEV 2500mg IV	CLZ 1mg + Placebo IV	Cessation within 15 min
Silbergleit et al. 2012 [25]	USA	RCT	893	43 (22) vs 44 (22)	250/448 (55.8%) vs 238/445 (53.5%)	CSE	MDZ 10mg IM	LZP 4mg IV	Cessation at ED arrival
Nakken et al. 2011 [26]	Norway	Prospective Cohort	22 (80 events)	42.4 (25–68)*	12/22 (54.5%)	Serial SE	MDZ 10–20mg Buccal	DZP 10–30mg Rectal	Cessation <10 min
De Haan et al. 2010 [27]	The Netherlands	Prospective Crossover	21 (124 events)	40.2	13/21 (61.9%)	Refractory SE	MDZ 10mg IN	DZP 10mg Rectal	Cessation <15 min
Fitzgerald et al. 2003 [28]	USA	Retrospective Cohort	6 (57 events)	29 (20–39)†	NR	GCSE (>10 min)	DZP 15–20mg Rectal	LZP 4mg IV	Cessation <10 min
Alldredge et al. 2001 [29]	USA	RCT	205	50.1 (19.1)	129/205 (62.9%)	GCSE	LZP 2mg/DZP 5mg IV	Placebo IV	Cessation at ED arrival

Abbreviations: BZD, benzodiazepine; CLZ, clonazepam; CSE, convulsive status epilepticus; DZP, diazepam; ED, emergency department; GCSE, generalized convulsive status epilepticus; IM, intramuscular; IN, intranasal; IV, intravenous; LEV, levetiracetam; LZP, lorazepam; MDZ, midazolam; NR, not reported; OHSE, out-of-hospital status epilepticus; RCT, randomized controlled trial; SE, status epilepticus; Tx, treatment. Notes:

* Mean (range).

† Median (range or IQR). Age presented as Mean (SD) unless otherwise noted. For multi-arm studies, values are presented as Intervention vs Comparator. Sample sizes reflect total enrolled; parenthetical values indicate events when studies used event-based analysis.

**Table 2:** Single-Arm Seizure Cessation Rates By Benzodiazepine Drug.

Drug	Study	Route	Dose	Events (n)	Total (N)	Rate (%)	95% CI (%)	Weight (%)	I^2^ (%)	τ^2^	Q (p-value)	Prediction Interval (%)
**Midazolam**	Silbergleit et al. 2012	IM	10mg	329	448	73.4	69.2–77.3	35.3	—	—	—	—
	Zitek et al. 2025	Mixed	Variable	393	484	81.2	77.5–84.4	35.8	—	—	—	—
	De Haan et al. 2010	IN	10mg	50	61	82.0	70.5–89.6	16.2	—	—	—	—
	Nakken et al. 2011	Buccal	10–20mg	32	43	74.4	59.8–85.1	12.8	—	—	—	—
	**Pooled (k=4, N=1036)**	—	—	804	1036	**77.6**	**72.1–82.7**	100.0	66.3	0.0096	8.91 (p=0.031)	60.5–91.0
**Diazepam**	De Haan et al. 2010	Rectal	10mg	56	63	88.9	78.8–94.5	25.5	—	—	—	—
	Nakken et al. 2011	Rectal	10–30mg	30	37	81.1	65.8–90.5	24.5	—	—	—	—
	Fitzgerald et al. 2003	Rectal	15–20mg	30	36	83.3	68.1–92.1	24.4	—	—	—	—
	Alldredge et al. 2001	IV	5mg (×2)	29	68	42.6	31.6–54.5	25.6	—	—	—	—
	**Pooled (k=4, N=204)**	—	—	145	204	**74.7**	**50.7–92.6**	100.0	92.4	0.2426	39.48 (p<0.001)	2.5–87.8
**Lorazepam**	Silbergleit et al. 2012	IV	4mg	282	445	63.4	58.8–67.7	40.1	—	—	—	—
	Alldredge et al. 2001	IV	2mg (×2)	39	66	59.1	47.0–70.1	34.4	—	—	—	—
	Fitzgerald et al. 2003	IV	4mg	20	21	95.2	77.3–99.2	25.5	—	—	—	—
	**Pooled (k=3, N=532)**	—	—	341	532	**71.2**	**54.7–85.2**	100.0	85.1	0.0748	13.39 (p=0.001)	9.0–98.2
**Clonazepam**	Navarro et al. 2016	IV	1mg	57	68	83.8	73.3–90.7	100.0	N/A	N/A	N/A	N/A

Abbreviations: CI, confidence interval; I^2^, heterogeneity statistic; IM, intramuscular; IN, intranasal; IV, intravenous; k, number of studies; N, total sample size; N/A, not applicable (single study); PI, prediction interval; Q, Cochran's Q statistic; τ^2^, between-study variance.

**Table 3:** Component-Based Meta-Analysis For Drug, Route, and Dose Effects on Seizure Cessation.

Section	Component	Contributing Arms	Total Patients (N)	Coefficient (β)	SE	Odds Ratio	95% CI	P-value
**Drug Effect**	Placebo (Reference)	1	71	0.00	—	1.00	—	—
	Midazolam	4	1036	2.547	0.296	12.76	7.14–22.81	<0.001
	Diazepam	4	204	2.067	0.333	7.90	4.11–15.17	<0.001
	Lorazepam	3	532	1.870	0.301	6.49	3.60–11.69	<0.001
	Clonazepam	1	68	2.962	0.432	19.35	8.30–45.11	<0.001
**Route Effect**	IV (Reference)	5	668	0.00	—	1.00	—	—
	IM	1	448	0.491	0.135	1.63	1.25–2.13	<0.001
	IN	1	61	0.988	0.337	2.69	1.39–5.20	0.003
	Buccal	1	43	0.542	0.353	1.72	0.86–3.43	0.125
	Rectal	3	136	1.205	0.250	3.34	2.04–5.44	<0.001
	Mixed	1	484	0.937	0.142	2.55	1.93–3.37	<0.001
**Dose Effect**	Per 5mg MDZ-equivalent increase	12	1840	−0.248	0.106	0.78	0.63–0.96	0.019
**Model Statistics**	Between-study variance (τ^2^)	—	—	0.2881	—	—	—	—
	Residual heterogeneity (I^2^)	—	—	88.7%	—	—	—	—
	Residual Q-statistic	—	—	88.80	df=10	—	—	<0.001
	Variance explained (R^2^)	—	—	5.8%	—	—	—	—

Abbreviations: β, regression coefficient (log-odds scale); CI, confidence interval; df, degrees of freedom; I^2^, heterogeneity statistic; IM, intramuscular; IN, intranasal; IV, intravenous; MDZ-eq, midazolam equivalents; N, total sample size; OR, odds ratio; R^2^, coefficient of determination; SE, standard error; τ^2^, between-study variance.

**Table 4:** Pairwise Drug Comparisons with Direct, Indirect, and Combined Estimates.

Comparison	Direct Evidence	Direct OR (95% CI)	Indirect Pathway	Indirect OR (95% CI)	Combined OR (95% CI)	Consistency P-value
Lorazepam vs Placebo	Alldredge et al. 2001	5.39 (2.54–11.44)	—	—	5.39 (2.54–11.44)	—
Diazepam vs Placebo	Alldredge et al. 2001	2.78 (1.32–5.85)	—	—	2.78 (1.32–5.85)	—
Lorazepam vs Diazepam	Alldredge et al. 2001	1.94 (0.98–3.86)	Via Placebo	1.94 (0.67–5.60)	1.94 (1.09–3.46)	1.000
Midazolam vs Lorazepam	Silbergleit et al. 2012	1.60 (1.20–2.12)	—	—	1.60 (1.20–2.12)	—
Midazolam vs Diazepam	De Haan et al. 2010; Nakken et al. 2011	0.62 (0.30–1.29)	Via Lorazepam	3.10 (1.48–6.53)	1.38 (0.82–2.33)	0.003
Midazolam vs Placebo	—	—	Via Lorazepam	8.62 (3.86–19.26)	8.62 (3.86–19.26)	—
Clonazepam vs Placebo	—	—	Single-arm comparison	19.35 (8.18–45.76)	19.35 (8.18–45.76)	—
Any BZD vs Placebo	Alldredge et al. 2001	3.85 (1.98–7.46)	—	—	3.85 (1.98–7.46)	—

Abbreviations: BZD, benzodiazepine; CI, confidence interval; OR, odds ratio.

**Table 5:** Pooled Safety Outcomes by Drug and Route.

Section	Study	Drug	Route	Events (n)	Total (N)	Rate (%)	95% CI (%)	Weight (%)	I^2^ (%)	Definition
**Respiratory Complications/Intubation**	Alldredge et al. 2001	Lorazepam	IV	7	66	10.6	5.2–20.3	—	—	Respiratory intervention
	Alldredge et al. 2001	Diazepam	IV	7	68	10.3	5.1–19.8	—	—	Respiratory intervention
	Silbergleit et al. 2012	Midazolam	IM	63	448	14.1	11.1–17.6	—	—	Intubation
	Silbergleit et al. 2012	Lorazepam	IV	64	445	14.4	11.4–17.9	—	—	Intubation
	Navarro et al. 2016	Clonazepam	IV	2	68	2.9	0.8–10.1	—	—	Respiratory disorder SAE
	Navarro et al. 2016	Clonazepam+LEV	IV	3	68	4.4	1.5–12.2	—	—	Respiratory disorder SAE
	Zitek et al. 2025	Midazolam	Mixed	7	598	1.2	0.6–2.4	—	—	Intubation
	Fitzgerald et al. 2003	Diazepam	Rectal	0	36	0.0	0.0–9.6	—	—	Respiratory difficulty
	Fitzgerald et al. 2003	Lorazepam	IV	0	21	0.0	0.0–15.5	—	—	Respiratory difficulty
	**Pooled**	**Midazolam**	—	70	1046	**6.1**	**0.0–24.4**	100.0	98.7	—
	**Pooled**	**Lorazepam**	—	71	532	**9.7**	**3.7–18.1**	100.0	70.8	—
	**Pooled**	**Diazepam**	—	7	104	**4.6**	**0.1–20.0**	100.0	83.6	—
	**Pooled**	**Clonazepam**	—	2	68	**2.9**	**0.8–10.1**	100.0	N/A	—
**Hypotension**	Silbergleit et al. 2012	Midazolam	IM	12	448	2.7	1.5–4.6	—	—	Not specified
	Silbergleit et al. 2012	Lorazepam	IV	13	445	2.9	1.7–4.9	—	—	Not specified
	Navarro et al. 2016	Clonazepam	IV	3	68	4.4	1.5–12.2	—	—	Circulatory failure
	Navarro et al. 2016	Clonazepam+LEV	IV	5	64	7.8	3.4–17.0	—	—	Circulatory failure
	**Pooled**	**All Active BZD**	—	33	1025	**3.5**	**2.2–5.2**	100.0	30.1	—
**Excessive Sedation**	De Haan et al. 2010	Midazolam	IN	40	59	67.8	55.1–78.3	—	—	Reported sedation
	De Haan et al. 2010	Diazepam	Rectal	34	62	54.8	42.5–66.6	—	—	Reported sedation
	Nakken et al. 2011	Midazolam	Buccal	9	43	20.9	11.4–35.2	—	—	Sedation >2h
	Nakken et al. 2011	Diazepam	Rectal	18	37	48.6	33.4–64.1	—	—	Sedation >2h
	**Pooled**	**Midazolam**	—	49	102	**44.0**	**6.3–86.6**	100.0	95.7	—
	**Pooled**	**Diazepam**	—	52	99	**52.5**	**42.7–62.2**	100.0	0.0	—
**Mortality**	Alldredge et al. 2001	Lorazepam	IV	5	65	7.7	3.3–16.8	—	—	In-hospital
	Alldredge et al. 2001	Diazepam	IV	3	67	4.5	1.5–12.4	—	—	In-hospital
	Alldredge et al. 2001	Placebo	IV	11	70	15.7	9.0–26.0	—	—	In-hospital
	Navarro et al. 2016	Clonazepam	IV	4	65	6.2	2.4–14.8	—	—	In-hospital
	Navarro et al. 2016	Clonazepam+LEV	IV	3	66	4.5	1.6–12.5	—	—	In-hospital
	Halliday et al. 2022	BZD	Mixed	2	35	5.7	1.6–18.6	—	—	In-hospital
	Halliday et al. 2022	No BZD	—	0	18	0.0	0.0–17.6	—	—	In-hospital
	**Pooled**	**All Active BZD**	—	17	298	**6.4**	**3.9–9.4**	100.0	0.0	—
**Comparative Safety** *(Active BZD vs Placebo)*	Alldredge et al. 2001	Respiratory	—	—	—	OR = 0.40	0.18–0.88	—	—	BZD protective (p=0.023)
	Alldredge et al. 2001	Mortality	—	—	—	OR = 0.35	0.13–0.91	—	—	BZD protective (p=0.031)

Abbreviations: BZD, benzodiazepine; CI, confidence interval; I^2^, heterogeneity statistic; IM, intramuscular; IN, intranasal; IV, intravenous; KET, ketamine; LEV, levetiracetam; N, total sample size; n, number of events; N/A, not applicable (single study); NNT, number needed to treat; OR, odds ratio; SAE, serious adverse event.

**Table 6:** Time-to-Cessation and Secondary Outcomes.

Section	Study	Drug	Route	N	Outcome	Value	95% CI	Notes
**Time to Seizure Cessation (minutes)**	Silbergleit et al. 2012	Midazolam	IM	329	Median (IQR)	3.3 (1.7–7.5)	—	Time from study drug
	Silbergleit et al. 2012	Lorazepam	IV	282	Median (IQR)	1.6 (0.9–4.7)	—	Time from study drug
	De Haan et al. 2010	Midazolam	IN	50	Mean ± SD	4.6 ± 3.4	3.7–5.5	Time to cessation
	De Haan et al. 2010	Diazepam	Rectal	56	Mean ± SD	4.3 ± 3.4	3.4–5.2	Time to cessation
	Nakken et al. 2011	Midazolam	Buccal	32	Mean (range)	5.0 (1–15)	—	Among responders
	Nakken et al. 2011	Diazepam	Rectal	30	Mean (range)	5.5 (1–15)	—	Among responders
	Alldredge et al. 2001	Lorazepam	IV	39	Mean ± SD	2.0 ± 1.5	1.5–2.5	Responders only
	Alldredge et al. 2001	Diazepam	IV	29	Mean ± SD	2.5 ± 2.0	1.8–3.2	Responders only
**Seizure Recurrence**	Alldredge et al. 2001	Lorazepam	IV	39	Events/N (%)	6/39 (15.4%)	7.2–29.7%	Recurrence <60 min
	Alldredge et al. 2001	Diazepam	IV	29	Events/N (%)	8/29 (27.6%)	14.7–45.7%	Recurrence <60 min
	Alldredge et al. 2001	Placebo	IV	15	Events/N (%)	3/15 (20.0%)	7.0–45.2%	Recurrence <60 min
	Silbergleit et al. 2012	Midazolam	IM	329	Events/N (%)	57/329 (17.3%)	13.6–21.8%	Additional Tx required
	Silbergleit et al. 2012	Lorazepam	IV	282	Events/N (%)	52/282 (18.4%)	14.3–23.4%	Additional Tx required
	Navarro et al. 2016	Clonazepam	IV	57	Events/N (%)	18/57 (31.6%)	21.0–44.5%	SE recurrence at 1h
	Navarro et al. 2016	Clonazepam+LEV	IV	54	Events/N (%)	14/54 (25.9%)	16.1–38.9%	SE recurrence at 1h
**Rescue Medication Required**	Silbergleit et al. 2012	Midazolam	IM	448	Events/N (%)	119/448 (26.6%)	22.7–30.8%	Second-line AED
	Silbergleit et al. 2012	Lorazepam	IV	445	Events/N (%)	163/445 (36.6%)	32.3–41.2%	Second-line AED
	De Haan et al. 2010	Midazolam	IN	61	Events/N (%)	11/61 (18.0%)	10.4–29.5%	Additional Tx
	De Haan et al. 2010	Diazepam	Rectal	63	Events/N (%)	7/63 (11.1%)	5.5–21.2%	Additional Tx
	Nakken et al. 2011	Midazolam	Buccal	43	Events/N (%)	11/43 (25.6%)	14.9–40.2%	Additional Tx
	Nakken et al. 2011	Diazepam	Rectal	37	Events/N (%)	7/37 (18.9%)	9.5–34.2%	Additional Tx
	Alldredge et al. 2001	Lorazepam	IV	66	Events/N (%)	27/66 (40.9%)	29.9–53.0%	Second BZD dose
	Alldredge et al. 2001	Diazepam	IV	68	Events/N (%)	39/68 (57.4%)	45.5–68.4%	Second BZD dose
	Alldredge et al. 2001	Placebo	IV	71	Events/N (%)	56/71 (78.9%)	68.0–86.8%	Second BZD dose
**Hospital Outcomes** (*ICU Admission)*	Silbergleit et al. 2012	Midazolam	IM	448	Events/N (%)	165/448 (36.8%)	32.5–41.4%	—
	Silbergleit et al. 2012	Lorazepam	IV	445	Events/N (%)	156/445 (35.1%)	30.8–39.6%	—
	Halliday et al. 2022	BZD	Mixed	35	Events/N (%)	25/35 (71.4%)	54.9–83.7%	—
	Halliday et al. 2022	No BZD	—	18	Events/N (%)	11/18 (61.1%)	38.6–79.7%	—
**Hospital Outcomes** (*Length of Stay [days])*	Silbergleit et al. 2012	Midazolam	IM	448	Median (IQR)	2 (1–5)	—	Days
	Silbergleit et al. 2012	Lorazepam	IV	445	Median (IQR)	2 (1–5)	—	Days
**Comparative Time Analysis**	Silbergleit et al. 2012	MDZ vs LZP	IM vs IV	611	Median difference	+1.7 min	—	MDZ slower post-drug
	De Haan et al. 2010	MDZ vs DZP	IN vs Rec	106	Mean difference	+0.3 min	−1.0 to +1.6	NS (p=0.65)
	Nakken et al. 2011	MDZ vs DZP	Buc vs Rec	62	Mean difference	−0.5 min	−2.2 to +1.2	NS (p=0.58)
	Alldredge et al. 2001	LZP vs DZP	IV vs IV	68	Mean difference	−0.5 min	−1.4 to +0.4	NS (p=0.26)

Abbreviations: AED, antiepileptic drug; BZD, benzodiazepine; Buc, buccal; CI, confidence interval; DZP, diazepam; ICU, intensive care unit; IM, intramuscular; IN, intranasal; IQR, interquartile range; IV, intravenous; LEV, levetiracetam; LZP, lorazepam; MDZ, midazolam; N, total sample size; NS, not significant; Rec, rectal; SD, standard deviation; SE, status epilepticus; Tx, treatment.

**Table 7:** Bias-Adjusted Target Trial Effect Analysis for Benzodiazepine Efficacy in Status Epilepticus.

Analysis / Parameter	k	Estimate	95% CI	τ^2^	I^2^	Interpretation
**PRIMARY ANALYSIS: POOLED EFFICACY ESTIMATES**						
Traditional random-effects	23	72.3%	68.1–76.1	0.111	73.3%	Unadjusted pooled seizure cessation rate
***Target trial effect (θ)*** *	**23**	**64.6%**	**57.6–71.1**	**0.065**	**28.4%**	**Bias-adjusted estimate for ideal double-blind RCT**
+ Route confounding adjustment	23	64.7%	57.5–71.2	0.067	29.0%	Additional adjustment for non-IV route comparisons
+ Sample size adjustment	23	65.8%	56.8–73.7	0.091	22.3%	Full model with small-study effect adjustment
**BIAS COEFFICIENTS (β): QUANTIFIED METHODOLOGICAL BIASES**						
Open-label design (vs double-blind)	—	β = 0.757*	0.01–1.51	—	—	Overestimates efficacy by OR × 2.13 (P = 0.048)
Observational design (vs RCT)	—	β = -0.104	−1.01–0.81	—	—	Non-significant (OR × 0.90, P = 0.812)
Route confounding present	—	β = -0.191	−0.85–0.47	—	—	Non-significant (OR × 0.83, P = 0.549)
Log sample size (centered)	—	β = -0.061	−0.33–0.21	—	—	OR × 0.94 per log-unit N (P = 0.646)
**HETEROGENEITY DECOMPOSITION:**						
Total I^2^ (unadjusted)	—	73.3%	—	—	—	Overall heterogeneity across all included studies
Explained I^2^ (by design features)	—	51.0%	—	—	—	Attributable to methodological differences
Residual I^2^ (true clinical variation)	—	22.3%	—	—	—	Remaining heterogeneity after bias adjustment
R^2^ (variance explained)	—	69.6%	—	—	—	Proportion of τ^2^ explained by design features
**DRUG-SPECIFIC TARGET TRIAL EFFECTS:**						
Midazolam	6	73.4%	66.2–79.5	0.171	0.0%	Traditional: 81.2%; Bias: +7.8 pp
Lorazepam	5	64.6%	42.8–81.7	0.570	0.0%	Traditional: 66.6%; Bias: +2.0 pp
Diazepam	9	59.3%	40.8–75.5	0.218	47.7%	Traditional: 70.7%; Bias: +11.4 pp
Clonazepam	1	—	—	—	—	Insufficient studies for bias- adjusted analysis
**VALIDATION: COMPARISON WITH STUDY DESIGN SUBSETS**						
Double-blind RCTs only	8	65.0%	55.8–73.1	0.153	82.1%	Closely approximates θ* (difference: +0.3%)
All RCTs (excluding observational)	20	72.5%	67.0–77.3	0.108	73.4%	Difference from θ*: +7.8 pp
Observational studies only	3	72.8%	56.1–84.8	0.156	80.8%	Difference from θ*: +8.1 pp
**95% Prediction interval for new ideal RCT**	—	—	**47.9–80.1**	—	—	**Expected range for a future ideal double-blind RCT**

Abbreviations: β = bias coefficient (log-odds ratio scale); CI = confidence interval; I^2^ = heterogeneity statistic; k = number of study arms; OR = odds ratio; pp = percentage points; RCT = randomized controlled trial; θ* = target trial effect (bias-adjusted estimate); τ^2^ = between-study variance. Notes:

* P < 0.05. Positive β values indicate the design feature leads to overestimation of efficacy compared to an ideal double-blind RCT. Target trial specification: Adults with status epilepticus (≥5 min), first-line benzodiazepine within 5 min, seizure cessation without recurrence within 10–20 min, double-blind RCT with adjudicated outcomes. Model: θ^j=θ*+Xj′β+uj+εj, where θ* = target trial effect, X_j_ = design feature deviations, β = bias coefficients. Estimation via REML with Knapp-Hartung adjustment.
