# Supplementary material for: Target Trial Emulation Meta-Analysis of Benzodiazepines For Out-of-Hospital Status Epilepticus in Adults
Source: Evidance Health Sci. Author manuscript; Available in PMC 2026 Apr 28. (PMC13120326; doi:10.65416/ehealthsci.2026.944313)
Supplement: 1 [file NIHMS2163193-supplement-1.pdf]

APPENDICES

Supplementary Table 1: Risk of Bias Assessment.

Study	Design	Tool	D1	D2	D3	D4	D5	D6	D7	Overall
Silbergleit 2012	Double-blind RCT (RAMPART)	RoB 2	Low	Low	Low	Low	Low	—	—	**Low**
Alldredge 2001	Double-blind RCT	RoB 2	Low	Low	Low	SC	Low	—	—	**Low**
Treiman 1998	Double-blind RCT (DVA)	RoB 2	Low	Low	SC	SC	Low	—	—	**SC**
Leppik 1983	Double-blind RCT	RoB 2	SC	Low	Low	SC	Low	—	—	**SC**
Appleton 1995	Open-label RCT	RoB 2	Low	SC	Low	SC	Low	—	—	**SC**
Chamberlain 1997	Open-label RCT	RoB 2	Low	SC	Low	SC	Low	—	—	**SC**
Dreifuss 1998	Open-label RCT	RoB 2	Low	SC	Low	SC	Low	—	—	**SC**
Lahat 2000	Open-label RCT	RoB 2	Low	SC	Low	SC	Low	—	—	**SC**
De Haan 2010	Open-label RCT	RoB 2	Low	SC	Low	SC	Low	—	—	**SC**
Nakken 2011	Open-label crossover RCT	RoB 2	Low	SC	Low	SC	Low	—	—	**SC**
Navarro 2016	Open-label RCT	RoB 2	Low	SC	SC	SC	SC	—	—	**SC**
Halliday 2022	Retrospective cohort	ROBINS-I	Ser	Mod	Low	Mod	Mod	Mod	Low	**Serious**
Zitek 2019	Prospective convenience	ROBINS-I	Ser	Mod	Low	Mod	Low	Mod	Low	**Serious**
Fitzgerald 2019	Retrospective cohort	ROBINS-I	Ser	Mod	Low	Mod	Mod	Mod	Low	**Serious**

RoB 2 Domains (D1–D5): D1=Randomization process; D2=Deviations from intended interventions; D3=Missing outcome data; D4=Measurement of the outcome; D5=Selection of the reported result. ROBINS-I Domains (D1–D7): D1=Confounding; D2=Selection of participants; D3=Classification of interventions; D4=Deviations from intended interventions; D5=Missing data; D6=Measurement of outcomes; D7=Selection of reported result. Judgments: Low=Low risk; SC=Some concerns; Mod=Moderate; Ser=Serious. Abbreviations: RoB 2, Cochrane Risk of Bias tool version 2; ROBINS-I, Risk Of Bias In Non-randomized Studies of Interventions; RCT, randomized controlled trial; DVA, Department of Veterans Affairs; RAMPART, Rapid Anticonvulsant Medication Prior to Arrival Trial.

Supplementary Table 2: Dose-Response Meta-Analysis by Drug.

Study	Drug	Route	Dose	N	Events	Rate (%)	MDZ-eq (mg)	Dose Category	OR per 5mg	95% CI	P-value	R^2
Silbergleit 2012	Midazolam	IM	10 mg fixed	448	329	73.4	10.0	Medium	—	—	—	—
De Haan 2010	Midazolam	IN	10 mg (≥25kg)	50	44	88.0	10.0	Medium	—	—	—	—
Nakken 2011	Midazolam	Buccal	10 mg fixed	43	34	79.1	10.0	Medium	—	—	—	—
Zitek 2019	Midazolam	IN	10 mg fixed	61	55	90.2	10.0	Medium	—	—	—	—
Lahat 2000	Midazolam	IN	0.2 mg/kg	23	20	87.0	5.0	Low	—	—	—	—
Chamberlain 1997	Midazolam	IM	0.2 mg/kg	12	9	75.0	5.0	Low	—	—	—	—
**Midazolam Summary**	—	—	**5.0–10.0 mg**	**637**	**491**	**77.1**	—	—	**0.68**	**0.11–4.23**	**0.701**	**0.041**
Silbergleit 2012	Lorazepam	IV	4 mg fixed	445	282	63.4	10.0	Medium	—	—	—	—
Alldredge 2001	Lorazepam	IV	2 mg (×2 max)	66	39	59.1	5.0	Low	—	—	—	—
Treiman 1998	Lorazepam	IV	0.1 mg/kg (max 4mg)	104	66	63.5	17.5	High	—	—	—	—
Appleton 1995	Lorazepam	IV	0.1 mg/kg	37	30	81.1	6.2	Medium	—	—	—	—
**Lorazepam Summary**	—	—	**2.0–7.0 mg**	**652**	**417**	**64.0**	—	—	**0.89**	**0.30–2.65**	**0.854**	**0.021**
Alldredge 2001	Diazepam	IV	5 mg (×2 max)	68	29	42.6	2.5	Low	—	—	—	—
De Haan 2010	Diazepam	Rectal	10 mg (≥25kg)	56	45	80.4	5.0	Low	—	—	—	—
Nakken 2011	Diazepam	Rectal	10 mg fixed	43	33	76.7	5.0	Low	—	—	—	—
Treiman 1998	Diazepam	IV	0.15 mg/kg (max 10mg)	89	52	58.4	5.2	Medium	—	—	—	—
Dreifuss 1998	Diazepam	Rectal	0.5 mg/kg	30	24	80.0	6.2	Medium	—	—	—	—
Appleton 1995	Diazepam	IV	0.3 mg/kg	36	24	66.7	3.8	Low	—	—	—	—
**Diazepam Summary**	—	—	**5.0–12.5 mg**	**322**	**207**	**64.3**	—	—	**2.65**	**1.08–6.49**	**0.101**	**0.531**
Navarro 2016	Clonazepam	IV	1 mg fixed	68	57	83.8	10.0	Medium	—	—	—	—
**Clonazepam Summary**	—	—	**1.0 mg**	**68**	**57**	**83.8**	—	—	—	—	—	—
**POOLED (All drugs)**	—	—	**2.5–17.5 mg MDZ-eq**	**1679**	**1172**	**69.8**	—	—	**1.10**	**0.72–1.67**	**0.670**	**0.012**

Equipotent Conversions: Midazolam 5mg = Lorazepam 2mg = Diazepam 10mg = Clonazepam 0.5mg. Dose Categories: Low (≤5mg MDZ-eq, k=6, N=304, 65.5%, 95% CI 60.0–70.6); Medium (5–10mg MDZ-eq, k=8, N=1271, 71.4%, 95% CI 68.8–73.8); High (>10mg MDZ-eq, k=1, N=104, 63.5%, 95% CI 53.9–72.1. Abbreviations: MDZ-eq, midazolam-equivalent; OR, odds ratio per 5mg increase; CI, confidence interval; R^2, coefficient of determination; IM, intramuscular; IN, intranasal; IV, intravenous.

Supplementary Table 3: Subgroup Analyses.

Subgroup Type	Subgroup	k	N	Events	Pooled (%)	95% CI	I² (%)	P-interaction
Study Design	RCT-DB	8	1298	861	65.0	57.5–71.9	82.3	0.011
	RCT-OL	12	431	346	79.0	74.8–82.6	0.0	—
	Observational	3	1437	1007	72.9	65.7–79.0	81.1	—
Route	IV	12	1024	669	68.3	61.4–74.5	73.5	0.024
	IM	2	460	338	73.4	69.2–77.2	0.0	—
	IN	3	134	119	87.9	81.2–92.4	0.0	—
	Buccal	1	43	34	78.4	63.9–88.2	0.0	—
	Rectal	3	129	102	78.4	70.5–84.6	0.0	—
	Mixed	2	1376	952	69.2	66.7–71.5	0.0	—
SE Definition	≥5 min	17	1942	1365	72.7	67.7–77.2	73.8	0.315
	≥10 min	2	193	118	61.0	54.0–67.6	0.0	—
	Clinical	4	1031	731	80.1	67.2–88.8	80.4	—
**OVERALL**	**All Studies**	**23**	**3166**	**2214**	**72.3**	**68.4–75.9**	**73.7**	**—**

***Methods:** Random-effects meta-analysis using logit transformation with DerSimonian-Laird estimator; P-interaction from chi-square test for subgroup differences (shown once per subgroup type). **Significant Interactions:** Study design (p=0.011) with open-label RCTs showing higher efficacy than double-blind; Route (p=0.024) with intranasal showing highest efficacy (87.9%). **Abbreviations:** k, number of study arms; N, total participants; CI, confidence interval; I², heterogeneity statistic; RCT-DB, double-blind randomized controlled trial; RCT-OL, open-label randomized controlled trial; IV, intravenous; IM, intramuscular; IN, intranasal; SE, status epilepticus.*

Supplementary Table 4: Sensitivity Analyses.

Analysis Type	Restriction	k	N	Events	Pooled (%)	95% CI	I² (%)	Δ vs Primary
Primary Analysis	All studies	23	3166	2214	72.3	68.4–75.9	73.7	Reference
Leave-One-Out	Excluding Alldredge 2001	21	3032	2146	73.9	70.3–77.2	66.0	+1.6%
	Excluding Appleton 1995	21	3093	2160	72.3	68.2–76.0	75.4	0.0%
	Excluding Chamberlain 1997	21	3142	2196	72.3	68.3–76.1	76.0	0.0%
	Excluding De Haan 2010	21	3060	2125	71.1	67.1–74.8	72.8	−1.2%
	Excluding Dreifuss 1998	22	3136	2190	72.1	68.1–75.8	74.5	−0.2%
	Excluding Fitzgerald 2019	22	2274	1602	73.1	68.6–77.2	74.8	+0.8%
	Excluding Halliday 2022	22	2682	1874	72.8	68.5–76.7	74.8	+0.5%
	Excluding Lahat 2000	21	3122	2177	71.8	67.7–75.5	74.9	−0.6%
	Excluding Leppik 1983	21	3088	2150	71.6	67.5–75.3	74.4	−0.8%
	Excluding Nakken 2011	21	3080	2147	71.9	67.8–75.7	75.3	−0.4%
	Excluding Navarro 2016	22	3098	2157	71.6	67.7–75.3	72.9	−0.7%
	Excluding Silbergleit 2012	21	2273	1603	73.3	68.8–77.4	72.5	+1.0%
	Excluding Treiman 1998	21	2973	2096	73.7	69.6–77.4	73.9	+1.4%
	Excluding Zitek 2019	22	3105	2159	71.3	67.5–74.9	71.3	−1.0%
Study Design	RCTs only	20	1729	1207	72.5	67.5–77.1	73.9	+0.2%
	Double-blind RCTs only	8	1298	861	65.0	57.5–71.9	82.3	−7.3%
Publication Year	2010 or later	10	2590	1831	74.7	70.2–78.7	77.1	+2.4%
	Before 2010	13	576	383	69.9	62.2–76.7	69.0	−2.4%
Sample Size	N ≥100 per arm	5	2373	1629	68.3	64.7–71.7	67.7	−4.0%
	N <100 per arm	18	793	585	75.8	69.0–81.5	75.4	+3.5%
Drug	Single BZD only (excl mixed)	21	1790	1262	73.7	68.6–78.2	76.0	+1.3%

***Methods:** Random-effects meta-analysis using logit transformation with DerSimonian-Laird estimator; Δ represents absolute change in pooled proportion compared to primary analysis. **Abbreviations:** k, number of study arms; N, total participants; CI, confidence interval; I², heterogeneity; Δ, change; RoB, risk of bias; RCT, randomized controlled trial; BZD, benzodiazepine.*

Supplementary Table 5: Network Consistency and Indirect Comparison Validation.

Comparison	Estimate Type	Source	k	N	OR	95% CI	P-value	I² (%)	P-consistency	Consistency Status
LZP vs Placebo	Direct	Alldredge 2001	1	137	5.24	2.49–11.01	<0.001	—	—	—
DZP vs Placebo	Direct	Alldredge 2001	1	139	2.72	1.30–5.69	0.008	—	—	—
LZP vs DZP	Direct	4 studies pooled	4	365	1.46	1.00–2.14	0.051	0.0	—	—
LZP vs DZP	Indirect	Via Placebo	2	276	1.92	0.68–5.48	0.221	—	—	—
LZP vs DZP	Combined	Direct + Indirect	6	641	1.51	1.05–2.16	—	—	0.630	Consistent
MDZ vs LZP	Direct	Silbergleit 2012	1	893	1.60	1.20–2.12	0.001	—	—	—
MDZ vs DZP	Direct	4 studies pooled	4	260	1.35	0.73–2.49	0.335	0.0	—	—
MDZ vs DZP	Indirect	Via LZP	5	1258	2.33	1.45–3.76	<0.001	—	—	—
MDZ vs DZP	Combined	Direct + Indirect	9	1518	1.90	1.30–2.76	—	—	0.167	Consistent
MDZ vs Placebo	Indirect	Via LZP	2	1030	8.36	3.77–18.52	<0.001	—	—	—
Any BZD vs Placebo	Direct	Alldredge 2001	1	205	3.75	1.95–7.23	<0.001	—	—	—
**Loop 1: LZP-DZP-Placebo**	Node-split	Direct vs Indirect	—	—	0.76*	—	—	—	0.630	Consistent
**Loop 2: MDZ-LZP-DZP**	Node-split	Direct vs Indirect	—	—	0.58*	—	—	—	0.167	Consistent
**Network Geometry**	Summary	4 nodes, 5 edges	11	2412	—	—	—	—	—	2 closed loops

***Network Structure:** Nodes = MDZ, LZP, DZP, Placebo; Direct edges = LZP-Placebo (k=1), DZP-Placebo (k=1), LZP-DZP (k=4), MDZ-LZP (k=1), MDZ-DZP (k=4); Closed loops = LZP-DZP-Placebo triangle, MDZ-LZP-DZP triangle. **Methods:** Direct estimates from head-to-head RCTs pooled using inverse-variance fixed-effects; indirect estimates via Bucher method; consistency assessed by comparing direct vs indirect log-OR with z-test (p<0.05 indicates significant inconsistency). **Node-Splitting Results:** *Values represent ratio of direct/indirect OR; Loop 1 (LZP-DZP-Placebo): direct OR 1.46 vs indirect OR 1.92, ratio 0.76, p=0.630 (consistent); Loop 2 (MDZ-LZP-DZP): direct OR 1.35 vs indirect OR 2.33, ratio 0.58, p=0.167 (consistent). **Key Findings:** Both closed loops showed no statistically significant inconsistency (p>0.05), supporting validity of indirect comparisons; however, MDZ vs DZP showed notable numerical discrepancy (direct OR 1.35 vs indirect OR 2.33) likely due to route confounding (direct studies compared non-IV MDZ vs rectal DZP; indirect pathway used IV routes); all BZDs significantly superior to placebo; LZP showed borderline superiority over DZP (combined OR 1.51, 95% CI 1.05–2.16); MDZ superior to LZP (OR 1.60, 95% CI 1.20–2.12). **Abbreviations:** k, number of studies; N, total participants; OR, odds ratio; CI, confidence interval; I², heterogeneity; MDZ, midazolam; LZP, lorazepam; DZP, diazepam; BZD, benzodiazepine; —, not applicable.*

Supplementary Table 6: Advanced Target Trial Analyses of Network Meta-Analysis and Probability Ranking.

Analysis / Parameter	k	N	Estimate	95% CI	I²	P-value	Interpretation
**BIAS-ADJUSTED NETWORK META-ANALYSIS:**							
Direct Comparisons							
Midazolam vs Lorazepam	1	893	OR = 1.60	1.20–2.12	—	0.001	MDZ significantly superior (RAMPART)
Lorazepam vs Diazepam	3	405	OR = 1.39	0.92–2.09	0.0%	0.119	Trend favoring LZP (non-significant)
Midazolam vs Diazepam	0	—	—	—	—	—	No direct comparison in adults
Indirect Comparison (Bucher Method)							
Midazolam vs Diazepam (via LZP)	4	1298	OR = 2.21	1.34–3.64	—	0.002	MDZ significantly superior to DZP
Network Estimates (Combined)							
**Midazolam vs Lorazepam**	**1**	**893**	**OR = 1.60**	**1.20–2.12**	**—**	**0.001**	**60% higher odds with MDZ**
**Lorazepam vs Diazepam**	**3**	**405**	**OR = 1.39**	**0.92–2.09**	**0.0%**	**0.119**	**39% higher odds with LZP (NS)**
**Midazolam vs Diazepam**	**4**	**1298**	**OR = 2.21**	**1.34–3.64**	**—**	**0.002**	**121% higher odds with MDZ**
League Table: OR (row vs column)							
MDZ vs LZP / MDZ vs DZP	—	—	1.60 / 2.21	—	—	—	MDZ reference
LZP vs MDZ / LZP vs DZP	—	—	0.63 / 1.39	—	—	—	LZP reference
DZP vs MDZ / DZP vs LZP	—	—	0.45 / 0.72	—	—	—	DZP reference
**COMPARATIVE EFFECTIVENESS PROBABILITY RANKING:**							
Probability of Being Best Drug							
**Midazolam: P(Best)**	**—**	**—**	**92.0%**	**—**	**—**	**—**	**Highest probability of being best**
Lorazepam: P(Best)	—	—	8.0%	—	—	—	Moderate probability
Diazepam: P(Best)	—	—	0.0%	—	—	—	Negligible probability
Probability of Being in Top 2							
Midazolam: P(Top 2)	—	—	99.9%	—	—	—	Near-certain top 2 placement
Lorazepam: P(Top 2)	—	—	94.2%	—	—	—	High probability top 2
Diazepam: P(Top 2)	—	—	5.9%	—	—	—	Unlikely top 2
Expected Rank (1 = Best)							
**Midazolam: E[Rank]**	**—**	**—**	**1.08**	**—**	**—**	**—**	**Expected rank ≈ 1st**
Lorazepam: E[Rank]	—	—	1.98	—	—	—	Expected rank ≈ 2nd
Diazepam: E[Rank]	—	—	2.94	—	—	—	Expected rank ≈ 3rd
P-Score (SUCRA Equivalent)							
**Midazolam: P-Score**	**—**	**—**	**95.9%**	**—**	**—**	**—**	**Highest ranking score**
Lorazepam: P-Score	—	—	51.1%	—	—	—	Intermediate ranking
Diazepam: P-Score	—	—	3.0%	—	—	—	Lowest ranking score
Rank Probability Distribution							
Midazolam: P(1st) / P(2nd) / P(3rd)	—	—	92.0% / 7.9% / 0.1%	—	—	—	Almost always ranks 1st
Lorazepam: P(1st) / P(2nd) / P(3rd)	—	—	8.0% / 86.1% / 5.8%	—	—	—	Usually ranks 2nd
Diazepam: P(1st) / P(2nd) / P(3rd)	—	—	0.0% / 5.9% / 94.1%	—	—	—	Almost always ranks 3rd
**FINAL EFFICACY RANKING (Bias-Adjusted):**							
**1. Midazolam (IM)**	**—**	**—**	**73.4%**	**69.1–77.4**	**—**	**—**	**Best efficacy; P-Score 95.9%**
**2. Lorazepam (IV)**	**—**	**—**	**64.2%**	**54.3–73.0**	**—**	**—**	**Second; P-Score 51.1%**
**3. Diazepam (IV)**	**—**	**—**	**61.4%**	**19.7–91.2**	**—**	**—**	**Third; P-Score 3.0%**

***Abbreviations:** CI = confidence interval; DB-RCT = double-blind randomized controlled trial; DZP = diazepam; E[Rank] = expected rank; I² = heterogeneity statistic; IM = intramuscular; IV = intravenous; k = number of study arms; LZP = lorazepam; MDZ = midazolam; N = total participants; NS = non-significant; OR = odds ratio; pp = percentage points; P-Score = probability score (SUCRA equivalent); θ* = target trial effect.*

Supplementary Table 7: Route-Stratified Target Trial Effect Analysis.

Analysis / Parameter	k	N	Estimate (%)	95% CI	τ²	I²	Interpretation
**OVERALL ADULTS ANALYSIS:**							
Traditional random-effects	12	2803	68.8	63.2–74.0	0.105	82.0%	Unadjusted pooled rate (adults only)
Target trial effect (θ)*	**12**	**2803**	**64.9**	**53.9–74.6**	**0.137**	**41.5%**	**Bias-adjusted estimate (adults only)**
95% Prediction interval	—	—	—	41.6–82.8	—	—	Expected range for new ideal adult RCT
Bias magnitude	—	—	+3.9 pp	—	—	—	Traditional overestimates by 3.9 percentage points
R² (variance explained)	—	—	49.4%	—	—	—	Design features explain half of heterogeneity
**INTRAVENOUS (IV) ROUTE:**							
IV Traditional random-effects	8	918	66.1	56.0–75.0	0.166	79.4%	Unadjusted IV pooled rate
IV Target trial effect (θ)*	**8**	**918**	**63.0**	**50.3–74.2**	**0.115**	**39.5%**	**Bias-adjusted IV estimate**
IV Bias magnitude	—	—	+3.1 pp	—	—	—	Open-label IV studies overestimate by 3.1 pp
β Open-label (IV)	—	—	1.075	−0.21–2.36	—	—	OR × 2.93 for open-label bias (P = 0.101)
**INTRAMUSCULAR (IM) ROUTE:**							
IM Traditional random-effects	2	509	81.9	0.4–100.0	0.503	85.4%	Unadjusted IM pooled rate
IM Target trial effect (θ)*	**2**	**509**	**66.2**	**—**	**0.000**	**0.0%**	**Bias-adjusted IM estimate (limited data)**
IM Double-blind RCT only (RAMPART)	1	448	73.4	69.1–77.4	—	—	Silbergleit 2012: high-quality reference
IM Bias magnitude	—	—	+15.7 pp	—	—	—	Observational IM study overestimates substantially
**MIXED/UNSPECIFIED ROUTE (Observational):**							
Mixed route (observational)	2	1376	69.2	51.6–82.5	0.000	0.0%	Halliday 2022 + Fitzgerald 2019
**IV ROUTE BY DRUG:**							
IV Lorazepam	4	652	64.2	54.3–73.0	0.047	37.6%	Treiman, Alldredge, Leppik, Silbergleit
IV Diazepam	3	198	61.4	19.7–91.2	0.235	86.5%	Treiman, Alldredge, Leppik
IV Clonazepam	1	68	83.8	72.7–91.4	—	—	Navarro 2016 (single open-label study)
**IM ROUTE BY DRUG:**							
IM Midazolam (all studies)	2	509	81.9	0.4–100.0	0.503	85.4%	Silbergleit 2012 + Zitek 2019
IM Midazolam (RCT only)	1	448	73.4	69.1–77.4	—	—	RAMPART trial only
**COMPARATIVE EFFECTIVENESS: IM vs IV (BIAS-ADJUSTED):**							
IV (double-blind RCTs only)	7	850	63.1	53.2–71.9	0.131	78.9%	Reference for bias-adjusted comparison
IM (double-blind RCT only)	1	448	73.4	69.1–77.4	—	—	RAMPART trial
**OR: IM vs IV**	**—**	**—**	**1.62**	**1.10–2.38**	**—**	**—**	**IM midazolam superior to IV (P = 0.015)**
Absolute difference (IM − IV)	—	—	+10.3 pp	—	—	—	IM shows 10.3% higher cessation rate
**RANKING BY DRUG-ROUTE COMBINATION (Bias-Adjusted):**							
1. IM Midazolam (RCT)	1	448	73.4	69.1–77.4	—	—	Highest efficacy (double-blind evidence)
2. IV Lorazepam	4	652	64.2	54.3–73.0	0.047	37.6%	Established IV standard
3. IV Diazepam	3	198	61.4	19.7–91.2	0.235	86.5%	High heterogeneity limits precision
4. IV Clonazepam	1	68	83.8†	72.7–91.4	—	—	Open-label only; likely overestimate

Abbreviations: CI = confidence interval; I² = heterogeneity statistic; IM = intramuscular; IV = intravenous; k = number of study arms; N = total participants; OR = odds ratio; pp = percentage points; RCT = randomized controlled trial; θ* = target trial effect (bias-adjusted estimate); τ² = between-study variance.

Supplementary Table 8: Publication Bias and Small-Study Effects Assessment.

Analysis	Test/Measure	Statistic	Value/Estimate	95% CI	P-value	Bias Detected	Interpretation
Overall (k=23)	Egger's regression	t-statistic	1.99	Intercept = 1.24	0.060	Yes	Funnel plot asymmetry detected
	Begg's rank correlation	Kendall's τ	0.371	—	0.014	Yes	Significant rank correlation
	Peters' test (binary)	t-statistic	1.29	Slope = 8.96	0.210	No	No small-study effects
Trim-and-Fill	Studies imputed	k_0	9	—	—	—	9 missing studies imputed
	Original estimate	Pooled rate	69.2%	67.5–70.8	—	—	Unadjusted estimate
	Adjusted estimate	Pooled rate	67.9%	66.2–69.5	—	—	Δ = −1.3% after adjustment
Funnel Plot Metrics	Pooled effect	Reference	69.2%	—	—	—	Central vertical line
	Studies left of mean	Count	7	—	—	—	Below pooled estimate
	Studies right of mean	Count	16	—	—	—	Above pooled estimate
	Asymmetry ratio (R/L)	Ratio	2.29	—	—	—	1.0 = perfectly symmetric
	Small study mean (N<median)	Efficacy	78.7%	—	—	—	Higher in small studies
	Large study mean (N≥median)	Efficacy	70.1%	—	—	—	Lower in large studies
	Small–large difference	Difference	+8.5%	—	—	—	Small-study effect present
Midazolam (k=6)	Egger's test	t-statistic	2.31	Intercept = 1.71	0.082	Yes	Asymmetry detected
	Begg's test	Kendall's τ	−0.067	—	1.000	No	No rank correlation
Lorazepam (k=5)	Egger's test	t-statistic	1.61	Intercept = 1.80	0.206	No	No asymmetry
	Begg's test	Kendall's τ	0.333	—	0.435	No	No rank correlation
Diazepam (k=9)	Egger's test	t-statistic	2.68	Intercept = 4.39	0.032	Yes	Asymmetry detected
	Begg's test	Kendall's τ	0.333	—	0.260	No	No rank correlation
Clonazepam (k=1)	—	—	—	—	—	—	Insufficient studies for analysis
**OVERALL ASSESSMENT**	**Bias Status**	—	—	—	—	**DETECTED**	**Egger p=0.060, Begg p=0.014**

Abbreviations: k, number of study arms; k_0, number of imputed studies; τ, Kendall's tau; CI, confidence interval; R/L, right-to-left ratio; N, sample size. Methods: Publication bias assessed using Egger's weighted regression test (regresses standardized effect on precision), Begg's rank correlation test (Kendall's τ between effect size and variance), and Peters' test (weighted regression on 1/N, recommended for binary outcomes); significance threshold α=0.10 for all tests; Duval and Tweedie's trim-and-fill method used to estimate number of missing studies and compute bias-adjusted pooled estimate; funnel plot asymmetry quantified by ratio of studies above vs below pooled estimate and comparison of small vs large study means (split at median sample size).
